# Probiotics, their action modality and the use of multi-omics in metamorphosis of commensal microbiota into target-based probiotics

**DOI:** 10.3389/fnut.2022.959941

**Published:** 2022-09-16

**Authors:** Maryam Idrees, Muhammad Imran, Naima Atiq, Rabaab Zahra, Rameesha Abid, Mousa Alreshidi, Tim Roberts, Abdelmuhsin Abdelgadir, Muhammad Khalid Tipu, Arshad Farid, Oluwaseyi Abraham Olawale, Shakira Ghazanfar

**Affiliations:** ^1^Department of Microbiology, Quaid-i-Azam University, Islamabad, Pakistan; ^2^National Agricultural Research Centre (NARC), National Institute for Genomics and Advanced Biotechnology (NIGAB), Islamabad, Pakistan; ^3^Department of Biotechnology, University of Sialkot, Sialkot, Pakistan; ^4^Department of Biology, College of Science, University of Hail, Hail, Saudi Arabia; ^5^Molecular Diagnostics and Personalized Therapeutics Unit, University of Ha’il, Ha’il, Saudi Arabia; ^6^Metabolic Research Group, Faculty of Science, School of Environmental and Life Sciences, The University of Newcastle, University Drive, Callaghan, NSW, Australia; ^7^Department of Pharmacy, Quaid-i-Azam University, Islamabad, Pakistan; ^8^Gomal Center of Biochemistry and Biotechnology, Gomal University, Dera Ismail Khan, Pakistan; ^9^Genomics Research Hub (GENOMAC HUB), Ogbomoso, Nigeria

**Keywords:** lactic acid bacteria, host-adapted strains, target-based probiotics, human probiotics formulation, multi-omics, bioprophylactics, biotherapeutics

## Abstract

This review article addresses the strategic formulation of human probiotics and allows the reader to walk along the journey that metamorphoses commensal microbiota into target-based probiotics. It recapitulates what are probiotics, their history, and the main mechanisms through which probiotics exert beneficial effects on the host. It articulates how a given probiotic preparation could not be all-encompassing and how each probiotic strain has its unique repertoire of functional genes. It answers what criteria should be met to formulate probiotics intended for human use, and why certain probiotics meet ill-fate in pre-clinical and clinical trials? It communicates the reasons that taint the reputation of probiotics and cause discord between the industry, medical and scientific communities. It revisits the notion of host-adapted strains carrying niche-specific genetic modifications. Lastly, this paper emphasizes the strategic development of target-based probiotics using host-adapted microbial isolates with known molecular effectors that would serve as better candidates for bioprophylactic and biotherapeutic interventions in disease-susceptible individuals.

## Introduction

The history of probiotics dates back over a 100 years, when Elie Metchnikoff, a Nobel laureate, theorized that “*health and longevity could be achieved by manipulating intestinal microflora, i.e., replacing harmful microbes with beneficial microbes”* (1–5). Since then, probiotics have gained substantial attention from researchers, clinicians, and the general public alike (6–8). The term probiotic originates from the Greek language and translates to “*for life*” (9).

**FIGURE 1:**
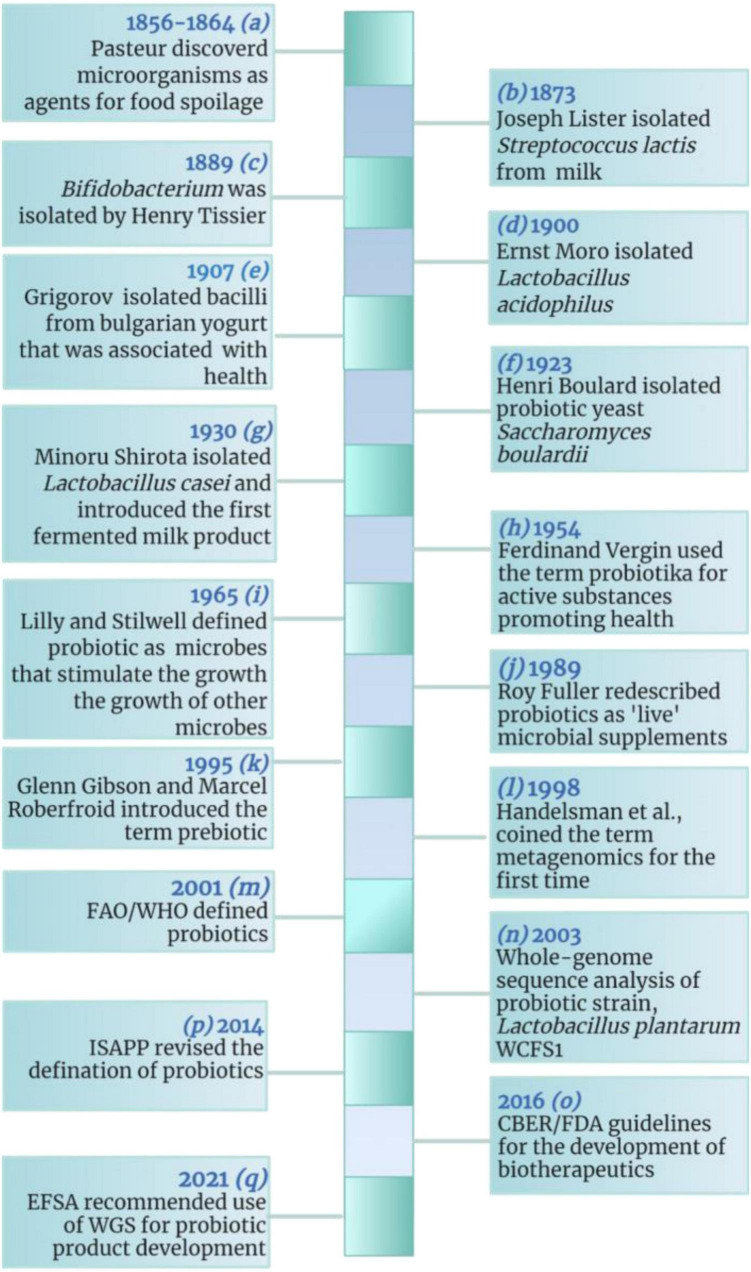
A brief history of probiotics. CBER, Center for Biologics Evaluation and Research; EFSA, European Food Safety Authority; FAO, Food and Agriculture Organization; FDA, Food and Drug Administration; ISAPP, International Scientific Association for Probiotics and Prebiotics; WHO, World Health Organization. The figure was drawn with BioRender.

A brief history of probiotics is given in Figure 1 (10–12). The evolution of probiotics began around 1856–1864 when French microbiologist Louis Pasteur proved that food spoilage was caused by microorganisms (13–15). Following the discovery of food spoilage agents, around 1873, British Scientist Joseph Lister isolated *Streptococcus lactis* (now known as *Lactococcus lactis*) from rancid milk (16–19). Meanwhile French pediatrician Henry Tissier isolated *Bacillus bifidum communis* (now known as *Bifidobacterium bifidum*) in 1889 (20) and observed that Bifidobacteria were dominant among the microflora of breast-fed infants (12, 21, 22). Shortly after the discovery of *Bifidobacteria*, around 1900 Austrian pediatrician Ernst Moro isolated a gram-positive bacterium and characterized it as *Bacillus acidophilus* (now known as *Lactobacillus acidophilus*) (23–25); while Bulgarian scientist Stamen Grigorov isolated *Lactobacillus bulgaricus* (aka *Lactobacillus delbrueckii* subsp. *bulgaricus*) from Bulgarian yogurt in 1907 (10, 26, 27). The year 1923 marked the isolation of the first probiotic yeast, *Saccharomyces boulardii* from lychee and mangosteen by French scientist Henri Boulard (28, 29); while in 1930, a Japanese scientist Minoru Shirota isolated *Lactobacillus casei* strain shirota (now known as *Lacticaseibacillus casei*) from the human intestine and developed the first probiotic fermented product called Yakult (30–32).

In 1954, German scientist, Ferdinand Vergin used the term *probiotika* to describe “*active substances that are essential for health”* and he also emphasized the adverse effects of antibiotics on useful intestinal microbiota (33, 34). Later, in 1965, American scientists Lilly and Stillwell coined the term *probiotic* to describe “*substances produced by one microorganism that stimulate the growth of another”* (35). While in 1974, Parker described probiotics as “*organisms and substances which contribute to intestinal microbial balance*” (36–38). Then probiotics were redefined by Roy Fuller by 1980 as, “*a live microbial feed supplement which beneficially affects the host by improving its intestinal microbial balance*” (10). The concept of prebiotics was introduced by English scientists Gibson and Roberfroid in 1995 (39–41), while American researchers Handelsman et al. coined the term metagenomics in 1998 (42). Later, in 2003, whole-genome of *Lactobacillus plantarum* WCFS1 (now *Lactiplantibacillus plantarum*) was sequenced and analyzed (32, 43).

In 2014, the International Scientific Association for Probiotics and Prebiotics (ISAPP), defined probiotics as “*live microorganisms that, when administered in adequate amounts, confer a health benefit on the host*” (44); while in 2016, Center for Biologics Evaluation and Research/Food and Drug Administration (CBER/FDA) produced guidelines for the development of live biotherapeutic products. Recently, European Food Safety Authority (EFSA) recommended the use of whole-genome sequence (WGS) analysis for microorganisms intended to be used in the food chain (45).

Despite the ever-increasing gamut of probiotic-based products, microbiome-targeted therapies, and associated literature, the efficacy of probiotics in many disease indications remains a conundrum. In the present article, we aim to address this issue by highlighting the importance of the host-adapted strains while strategically formulating probiotics intended for human use.

## How do probiotics exert their beneficial effects?

The action modality of probiotics is regulated by intricate and extensive molecular mechanisms that have not been well documented. However, various mechanisms have been hypothesized to explain the beneficial effects rendered by probiotics (46–49) including improvement of digestion, inhibition of pathogenic bacteria in the gastrointestinal tract, reduction of blood pressure and high blood glucose levels, improvement of intestinal health, reduction in serum cholesterol levels, toxin degradation, production of cofactors and vitamins, upregulation of the immune system, anti-inflammatory potential, and prevention of tumors and cancers (6–8, 11, 31, 46, 50–54). Probiotics-mediated molecular strategies involved in promoting health-associated phenotype and alleviating disease-associated phenotype are summarized in Tables 1, 2, respectively.

**TABLE 1:** Summary of probiotics-mediated molecular mechanisms that promote health-phenotype.

Probiotic effect	Probiotic strain	Molecular mechanisms	References
Competition for nutrients and prebiotics	Unidentified probiotic	Compete for monomeric glucose against *C. difficile*	(59)
	*Bifidobacterium adolescentis* S2-1	Compete for vitamin K and other growth factors against *Porphyromonas gingivalis*	(60)
	*L. delbrueckii* and *L. acidophilus*	Readily deplete ferric hydroxide to restrict pathobionts and pathogens	(61)
Production of antimicrobials	*Bifidobacteria* and *Lactobacilli*	Produce bacteriocins and other antimicrobials	(62)
	*Bifidobacterium longum* subsp. *infantis* 157F	Produce acetate against *E. coli O157:H7* shiga toxin	(66)
	*Lactobacillus reuteri*	Produce antimicrobials against *Staphylococcus aureus*	(67)
	*L. pentosus*, *L. rhamnosus* I, *L. paracasei* subsp. *paracasei*, *L. delbrueckii* subsp. *lactis* I and *Streptococcus uberis* II	Produce BLISs against clinical isolates of *Candida albicans* and non-*Candida albicans*	(68)
	*Lactobacillus sakei* JD10	Produce carnocin to impede the growth of lung pathogens	(69)
	*Lactiplantibacillus plantarum* PUK6	Produce plantaricin NC8, A, and EF	(70)
	*Bacillus paranthracis* MHSD3	Produce BLISs against *Staphylococcus aureus, S. epidermidis, S. saprophyticus*, and *Escherichia coli*	(71)
Bioconversion of substrates	*Bifidobacterium animalis* subsp. *lactis* HN019, *Lactobacillus. reuteri* ATCCPTA5289, and *L reuteri* DSM17938	Bioconversion of host’s substrates to alleviate chronic periodontitis	(74–78)
	*Saccharomyces boulardii* and *Saccharomyces cerevisiae*	Biosynthesize selenomethionine and selenocysteine from selenate	(79, 80)
	*Streptococcus thermophilus* ST8, *Enterococcus faecium* ST3, *Lactiplantibacillus plantarum* LP6 & LP9, *Lactobacillus acidophilus* LP16-2, *Bifidobacterium animals* ST20	Produce of γ-aminobutyric acid (GABA)	(81)
Production of growth-promoting substrates	*Lactiplantibacillus plantarum*	Produce folate from para-aminobenzoic acid (pABA)	(84)
	*B. adolescentis*, and *B. pseudocatenulatum*	Produce folate	(85)
	*Lactobacillus delbrueckii* subsp. *bulgaricus, L. mucosae*, *L. fermentum*, and *L. plantarum* CRL 2130	Biosynthesize of riboflavin	(86, 91)
Auto-aggregation, biofilm formation, and co-aggregation	*Enterococcus faecium* C4, *Enterococcus faecium* F6, *Pediococcus pentosaceus* C3, *Pediococcus pentosaceus* C6, and *Pediococcus pentosaceus* C8	Form Biofilm Auto-aggregate Co-aggregate with *Bacillus cereus, Escherichia coli, and Salmonella typhimurium*	(109)
	*Streptococcus salivarius* K12	Auto-aggregate Co-aggregate and disrupt biofilm *C. albicans* ALC3, ALC2, ATCC MYA-4901	(111)
	*Saccharomyces boulardii*	Auto-aggregate Co-aggregate with *C. glabrata* ZIM 2369, *C. krusei* ATCC 6258, *C. albicans* ATCC 10261, and *C. glabrata* ZIM 2382	(112)
	*Levilactobacillus gastricus* BDUMBT09, *L. brevis* BDUMBT11, *L. brevis* BDUMBT08, *L. casei* BDUMBT13, and *L. casei* BDUMBT12	Auto-aggregate Co-aggregate with *Shigella flexneri* and *Enterococcus faecalis*	(113)
	*Streptococcus salivarius* CP163 and *S. salivarius* CP208	Auto-aggregate co-aggregate with *Salmonella enteritidis* and *E. coli*	(114)
	*Lactiplantibacillus plantarum* ATCC 1058	Co-aggregate with *Pseudomonas aeruginosa* or *Staphylococcus aureus*	(115)
	*Lactobacillus curvatus BSF206* and *Pediococcus pentosaceus AC1-2*	Auto-aggregate, co-aggregate and disrupt biofilm formation of carcinogenic *S. mutans* ↓ *ftf*, *brpA, gtfA*, and *gtfB*	(116)
Maintenance of barrier integrity	*Lactobacillus rhamnosus* strain GG and *Lactobacillus plantarum* 299v	prevent adherence of *Escherichia coli* to human colon cells	(125)
	*L. plantarum*	Produce HYA ↑ ZO-1, occludin, claudin-1 ↓ TJ permeability	(127)
	*Bifidobacterium dentium* N8	↑ TEER, ZO-1, occludin, claudin ↓IL-1β, IL-6, TNF-α.	(128)
	*Lactobacillus* strains	↑ TEER, ZO-1, occludin, claudin ↓ TJ permeability, IL-8, NF-κB	(129)
Immune modulation	*Apilactobacillus kosoi* 10HT, *A. kunkeei* JCM16173T, and *A. apinorum* JCM30765T	*↑ IgA induction* *LTA contains dihexosyl glycerol*	(95)

**TABLE 2:** Summary of probiotic-mediated molecular mechanisms that alleviate disease-phenotype.

Disease/indication	Probiotic strain	Action modality	References
COVID-19	*Bifidobacterium longum BB536, Lactobacillus plantarum SNK12*, and *Lactococcus lactis* subsp. *lactis* LLL970	Innate cytokine index IL-6 and other dysregulatory cytokines	(93)
Asthma	*Lactobacillus acidophilus* LA-5, *L. rhamnosus* GG, and *Bifidobacterium animalis* subspecies *lactis* BB-12 with prebiotics	↓ CCL11, PI3K/Akt, TLR4/NF−κB ↓ IL-4, IL-5, IL-13, IL-17, IL-25, IL-33 ↑ IFN-γ ↓ IgE, OVA-specific IgE, IgG1 ↓ Cys-LTs, LTC4, LTB4	(97)
	*Bifidobacterium breve* 207-1 and *Lacticaseibacillus paracasei* 207-27	↓ IgE, IgG3 ↑ TNF-α, IL-6, IL-10, IL-12 ↑ SCFAs	(98)
	Lacticaseibacillus paracasei MG4272, MG4577, and MG4657 and Lactobacillus gasseri MG4247	↓ IL-4, IL-5, IL-13 ↓ STAT-6	(99)
Burn wounds	*Lactiplantibacillus plantarum* ATCC 1058	Produce antimicrobials and co-aggregate with *Pseudomonas aeruginosa* or *Staphylococcus aureus*	(115)
Chronic periodontitis	*Bifidobacterium animalis* subsp. *lactis* HN019, *Lactobacillus. reuteri* ATCCPTA5289, and *L reuteri* DSM17938	Attenuation	(74–78)
NEC	*Bifidobacterium longum* subsp. *infantis* ATCC 15697	Indole-3-lactic acid ↓ IL-1β, IL-8	
IBD	Riboflavin-producing probiotic phenotype	Prevention or treatment	(89, 90)
	*Bifidobacterium bifidum* BGN4	↑ TJ proteins, FOXP3 ↓ IL-1β, COX-2, T-bet, IκB-α	(137)
	*Pediococcus pentosaceus* CECT 8330, *Lactobacillus acidophilus* ATCC 4356	↑ TJ proteins ↓ IL-1β, IL-8, NF-κB	(138, 139)
CKD	*Lactobacillus rhamnosus* 34	↓ GDUT: TMAO, IS ↓ serum creatinine, proteinuria ↓ TNF-α, IL-6, IL-8, NF-κB ↑ TEER	(140)
CKD-linked CVD	*Bifidobacterium bifidum* BGN4 and *Bifidobacterium longum* BORI	↑ SCFAs ↓ IL-6, calprotectin ↓ CD14^+^ and CD16^+^ ↑ CD4^+^ and CD25^+^	(144)
Oral cancer	*Streptococcus salivarius* K12	Disrupt the dimorphism, aggregation, and biofilm formation of oral cancer isolates *C. albicans*	(111)
	*Lactobacillus curvatus BSF206* and *Pediococcus pentosaceus AC1-2*	Auto-aggregate, co-aggregate and disrupt biofilm formation of carcinogenic *S. mutans* by downregulating the expression of *ftf*, *brpA, gtfA*, and *gtfB*	(116)
Cancer	*Streptococcus salivarius* CP163 and *S. salivarius* CP208	Auto-aggregate and co-aggregate with *Salmonella enteritidis* and *E. coli*	(114)
Mucositis	*L. plantarum* CRL 2130	Prevention	(91)

### Competition for nutrients and prebiotics

One of the mechanisms imparted by probiotics to maintain health is to compete for nutrients that would otherwise be consumed by enteropathogenic microorganisms (55–57). Strain et al. reported that, unlike the distantly related species, the commensal symbionts and their phylogenetically related pathobionts often compete for similar microbial resources. These metabolites include vitamins, trace elements, carbon substrates, and secondary bile acids (58). *Clostridium difficile* is a potentially pathogenic bacteria that rely on monosaccharides for nutrition and growth. A study investigated the colonization resistance of *C. difficile* in a continuous flow culture model colonized with the mouse cecal flora. It was reported that an unidentified organism was highly efficient in taking up the monomeric glucose molecules, N-acetylneuraminic acid, and N-acetylglucosamine, thus limiting the growth of *C. difficile* (59). Similarly, *Bifidobacterium adolescentis* S2-1 outcompetes *Porphyromonas gingivalis* by readily utilizing vitamin K and other growth factors thus averting infection through nutrient limitation (60).

Augmenting the research mentioned above, Elli et al. reported the role of *Lactobacilli* in iron acquisition. Iron constitutes one of the micronutrients fundamental for the growth and functioning of most bacteria. Probiotic bacteria like *L. delbrueckii* and *L. acidophilus* bind ferric hydroxide at their cell surface, rendering thus, iron supply unavailable for pathobionts and pathogens (61).

### Production of antimicrobials

Another possible mechanism employed by probiotics for maintaining the health phenotype is the synthesis of antimicrobial compounds (62). Different species of *Bifidobacteria* and *Lactobacilli* produce various types of bacteriocins and other antimicrobial compounds that inhibit the proliferation of pathogens. Bacteriocins are defined as “compounds produced by bacteria that have a biologically active protein moiety and a bactericidal action” (63); these antibacterial peptides are small, cationic, and comprised of 30–60 amino acid molecules (64). Other biologically active antimicrobial compounds synthesized by Lactic acid bacteria (LAB) to inhibit the harmful microflora include short-chain fatty acids (SCFAs), diacetyl, and hydrogen peroxide (46, 65). SCFAs produced by gut microbes have long been associated with health benefits to the host. The commensal microbiota metabolizes indigestible carbohydrates from high-rich fiber diets into SCFAs. It has been reported elsewhere (66) that *Bifidobacterium longum* subsp. *infantis* 157F produces acetate that plays a significant part in the defense surveillance of the host intestinal epithelial cells (IECs). It prevents the translocation of E. coli O157:H7-induced shiga toxin from the gut lumen to blood thereby, protecting the host against the *E. coli O157:H7* shiga toxin-induced death. Furthermore, an ATP-binding-cassette (ABC)-type promoter that facilitates the uptake of carbohydrates has been found that upregulates the acetate production by *bifidobacteria* strains (66).

*Lactobacillus reuteri* has been reported to protect keratinocytes by producing antimicrobials to restrict the growth of *Staphylococcus aureus* (67). Similarly, *L. pentosus*, *L. rhamnosus* I, *L. paracasei* subsp. *paracasei*, *L. delbrueckii* subsp. *lactis* I, and *Streptococcus uberis* II are reported to produce bacteriocin-like inhibitory substances (BLISs) against clinical isolates of *Candida albicans* and non-*Candida albicans* from women with vulvovaginitis (68). While whole-genome sequence analysis of a lung isolate *Lactobacillus sakei* JD10 reports a gene encoding bacteriocin carnocin that inhibit the proliferation of pathogenic bacteria (69). Another interesting study reported that *Lactiplantibacillus plantarum* PUK6 produces multiple bacteriocins including plantaricin NC8, A, and EF (70); while Diale et al. reported the production of multiple BLISs from *Bacillus paranthracis* MHSD3 with anti-proliferative potential against *Staphylococcus aureus, S. epidermidis, S. saprophyticus*, and *Escherichia coli* (71).

### Bioconversion of substrates

Another interesting feat of probiotic microorganisms is their ability to modify substrates to the host’s benefit (46–49). Probiotics outcompete enteropathogens like *Salmonella typhi, Helicobacter pylori, Entamoeba histolytica, Clostridium difficile*, and *E. coli* by lowering the gut pH, this is brought by fermentation of substrates that result in the production of a large variety of organic acids and volatile fatty acids (72). The probiotic-mediated bioconversion (PMB) of metabolites is reported to have antimicrobial, anticancer, anti-inflammatory, and antioxidant activities. Furthermore, recent studies reported the positive effects of PMB of metabolites in periodontitis (73), the use of probiotic *Bifidobacterium animalis* subsp. *lactis* HN019, *Lactobacillus reuteri* ATCCPTA5289, and *L. reuteri* DSM17938 [now *Limosilactobacillus reuteri* (32)] alleviated chronic periodontitis (74–78).

Another interesting example of probiotic bioconversion is the biosynthesis of seleno amino acids from inorganic selenium. *Saccharomyces boulardii* and *Saccharomyces cerevisiae* are reported to metabolize selenate into selenomethionine and selenocysteine (79, 80). While other recent studies reported probiotic candidates including *Streptococcus thermophilus* ST8, *Enterococcus faecium* ST3, *Lactiplantibacillus plantarum* LP6 & LP9, *Lactobacillus acidophilus* LP16-2, *Bifidobacterium animals* ST20 as γ-aminobutyric acid (GABA) producing strains (81), and the use of probiotics for the biodegradation of mycotoxins (82).

### Production of growth-promoting substrates

The probiotic microorganisms impart several health benefits to the host including the production of growth-promoting substrates (6–8, 11, 31, 46). Folate is required for DNA methylation, repair, and division, while its deficiency results in serious disorders (83). *Lactiplantibacillus plantarum* is reported to produce folate in the presence of para-aminobenzoic acid (pABA) (84) and another study reported the consumption of *Bifidobacteria* strains, *B. adolescentis*, and *B. pseudocatenulatum* resulted in a significant increase of folate concentration in fecal samples from 23 healthy individuals (85). Moreover, Liu et al. reported the potential of *Latilactobacillus sakei* LZ217 for folate production (83).

Riboflavin plays a crucial role in numerous cellular metabolic processes *via* its participation in oxidation-reduction reactions (86); its active forms flavin adenine dinucleotide (FAD) and flavin mononucleotide (FMN) are used in pharmaceutical, feed, and food industries. LAB strains harboring the potential to produce riboflavin have been reported (87). A study reported screening of 60 *Lactobacilli* isolates for the presence of riboflavin biosynthesis genes through a polymerase chain reaction (PCR)-based technique; the *Lactobacillus delbrueckii* subsp. *bulgaricus, L. mucosae, L. fermentum*, and *L. plantarum* carried a complete set of riboflavin structural genes, while the other isolates carried partial genes or the absence of related genes. The *Lactobacilli* carrying a partial set of riboflavin structural genes were unable to grow in the riboflavin-deficient culture medium (86). In a separate study, a trinitrobenzene sulfonic (TNBS)-induced mouse model was used to study the effect of soy milk fermented with riboflavin-producing *L. plantarum* CRL 2130. It dramatically reduced TNBS-induced intestinal damage (88). Inflammatory bowel diseases (IBD) could be prevented or treated using the Riboflavin-producing phenotype (89, 90). Furthermore, the *L. plantarum* CRL 2130 could be beneficial in preventing mucositis during cancer treatment while having no effect on the primary treatment (91).

### Immune stimulation

Immune stimulation is another aspect of probiotic modality; probiotics elicit immunomodulatory activity by downregulating inflammatory responses, activation of NK (Natural Killer) cells and DCs (Dendritic cells), modulating the expression of TLRs (Toll-like Receptors), secretion of specific Immunoglobulin A (IgA), regulating the proliferation of lymphocytes, and balancing the ratio of T-helper (Th1/Th2) cells (92). While the probiotic-induced immunomodulatory mechanisms are being discerned, their use as a biotherapeutic in preclinical and clinical trials is being evaluated. One such study evaluated the immunomodulatory potential of *Bifidobacterium longum BB536, Lactobacillus plantarum SNK12*, and *Lactococcus lactis* subsp. *lactis* LLL970 against COVID-19 using *in vitro* cytokine response assay followed by a single-arm, double-blind, prospective trial. Out of the three tested probiotics, *L. plantarum SNK12* significantly increased the innate cytokine index and decreased the plasma levels of IL-6, a major constituent responsible for complex immune dysregulation in COVID-19 patients. The study concluded that *L. plantarum* SNK12 mimics the blood cytokine profile present in early viral infection and demonstrates a high immunomodulatory ability and could be taken as a bioprophylactic for COVID-19 (93).

The structural components of LAB are of great interest for bioprophylactics and biotherapeutic approaches as they exert immunostimulatory effects and can be used in lieu of antibiotics, as vaccine adjuvants, or as cognitive enhancements (94). The IgA inducing potential of *Apilactobacillus kosoi* 10HT was compared to twenty-nine other LAB strains using murine Peyer’s patch cells as a model elsewhere (95). It was observed that the species belonging to the genus *Apilactobacillus* (*A. kosoi* 10HT, *A. kunkeei* JCM16173T, and *A. apinorum* JCM30765T) displayed significantly higher IgA-inducing potential than the other LAB isolates. Subsequently, lipoteichoic acids (LTAs) were purified from the three immunostimulatory *Apilactobacillus* strains, and their IgA-inducing potential was compared with LTAs from *Lacticaseibacillus rhamnosus* GG and *Lactiplantibacillus plantarum* JCM1149T; while the significance of LTA lies in the fact that it is an important immunostimulatory constituent of Gram-positive bacterial cell wall as it interacts with host’s PRRs (Pattern Recognition Receptors) and elicits an immune response. The results disclosed that LTAs from *Apilactobacillus* spp. had significantly higher immunostimulatory potential than the other two probiotic strains (*L. rhamnosus* GG and *L. plantarum* JCM1149T). Later, structural analysis of the LTA glycolipid *via* matrix-assisted laser desorption ionization–time-of-flight mass spectrometry (MALDI-TOF MS) revealed that LTA from *A. kosoi* 10HT carried dihexosyl glycerol, whereas other LAB strains carried trihexosyl glycerol. This dissimilarity in LTA structure could be linked to different IgA-inducing capacities (95).

As probiotics and prebiotics have immunomodulatory effects recent research is investigating their potential to restore asthma associated-immune system dysregulation and alleviate allegro-inflammatory reactions (96). One such study investigated the activity of *Lactobacillus acidophilus* LA-5, *L. rhamnosus* GG, and *Bifidobacterium animalis* subspecies *lactis* BB-12 in regulating the toll-like receptor 4/nuclear factor-kappa light chain enhancer of activated B cells (TLR4/NF-κB) pathway and acute airway inflammation (97). The prebiotics used were FOS (fructo-oligosaccharides) and GOS (galacto-oligosaccharides) and the allergic asthma model of BALB/c mice was used. The results indicated that probiotics have the potential to downregulate the gene expression of the Chemokine (C-C motif) ligand (CCL11) and TLR4. Probiotics also controlled airway hyperresponsiveness, levels of immunoglobulins, eosinophil infiltration to perivascular and bronchoalveolar lavage fluid (BALF), Glutamic pyruvic transaminase, interleukin-17 (IL-17), mucus secretion, Eosinophil peroxidase activity (EPO), and goblet cell hyperplasia. While probiotics and prebiotics regulated gene expression of AKT (Protein kinase B), MUC5a (Mucin 5a), levels of cytokines (IL-4, 5, 13, 25, and 33), leukotrienes, NLRP3 (NOD-like receptor family pyrin domain containing 3), peribronchial inflammation, MyD88 (Myeloid differentiation primary response 88), NF-κB and increase in IL-38 gene expression. Whereas prebiotic controlled PI3K (Phosphoinositide 3-Kinase) gene expression. In short, the study concluded that probiotics-prebiotics-induced tolerance could attenuate allegro-inflammatory reactions (97).

Liang et al. investigated the anti-allergy effect of two probiotic strains *L. paracasei* 207-27 and *B. breve* 207-1 compared with *L. rhamnosus GG* using an allergic mouse model. It was seen that *L. paracasei* 207-27 possess a higher potential for downregulating serum immunoglobulin E (IgE) levels than *L. rhamnosus* GG while *B. breve* 207-1 displayed a weak anti-allergic effect. Furthermore, both probiotic strains reduced serum immunoglobulin G3 (IgG3) levels and induced tumor necrosis factor-alpha (TNF-α), IL-6, IL-10, and IL-12. In addition, *L. paracasei* 207-27 remarkedly upregulated the level of cecal SCFAs in allergic mouse models (98). Another recent study reported the potential of Lacticaseibacillus paracasei MG4272, MG4577, and MG4657 and Lactobacillus gasseri MG4247 to attenuate allergic inflammation by downregulating the expression of IL-4, IL-5, IL-13, and STAT6 phosphorylation (99).

### Auto-aggregation, biofilm formation, and co-aggregation

The natural attachment and immobilization of microorganisms to abiotic or biotic surfaces in a submerged environment is termed as a biofilm. The presence of a high density of microbial cells in a biofilm ensures that microbes can withstand challenges such as osmotic stress, antibiotic stress, pH change, or starvation (100–105). LAB have been shown to form biofilms while displaying antagonistic activities against a variety of foodborne pathogens (106, 107); auto-aggregation of the bacterial cells and co-aggregation of bacteria with pathogens play a significant role in preventing pathogens from infecting, adhering to, and colonizing host cells, especially in the gastrointestinal tract (108). A study exploited the potential role of LAB biofilms and their naturally secreted compounds to control the biofilms of foodborne pathogens (109). It reported that isolates *Enterococcus faecium* C4, *Enterococcus faecium* F6, *Pediococcus pentosaceus* C3, *Pediococcus pentosaceus* C6, and *Pediococcus pentosaceus* C8 could auto-aggregate as well as co-aggregate with *Bacillus cereus, Escherichia coli, and Salmonella typhimurium*. Among all the tested strains, *Enterococcus faecium* C4 developed a significant amount of exopolysaccharide (EPS) and a biofilm with the highest cell density (109). It has been previously reported elsewhere (110) that EPS facilitates the colonization of probiotic bacteria on the intestinal epithelium.

In a study investigated the effect of probiotic *Streptococcus salivarius* K12 on pathogenic *Candida albicans* strains including oral cancer isolate ALC3, an acquired immunodeficiency syndrome (AIDS) isolate ALC2, and *C. albicans* ATCC MYA-4901. It was revealed that the auto-aggregation of *C. albicans* strains was categorized as high, whereas co-aggregation of *C. albicans* strains with *S. salivarius* K12 was categorized as low. *S. salivarius* K12 also disrupted the biofilm formation of the pathogenic yeast strains, evident by a significant decrease in the total cell count of *C. albicans* when co-cultured with *S. salivarius* K12 as compared with monocultured *C. albicans*. So, *S. salivarius* K12 efficiently disrupted the dimorphism, aggregation, and biofilm formation of *C. albicans* (111). In a separate study, probiotic yeast *Saccharomyces boulardii* and pathogenic *Candida* strains including *C. glabrata* ZIM 2369, *C. krusei* ATCC 6258, *C. albicans* ATCC 10261, and *C. glabrata* ZIM 2382 were evaluated for their potential to auto-aggregate and co-aggregate. It was revealed that all yeast strains harbored the potential to auto-aggregate and it increased significantly after 24 h of incubation, while co-aggregation between the probiotic yeast and the pathogenic yeasts was subjected to strain-specificity. However, it was indisputable that *Saccharomyces boulardii* significantly impeded the aggregation of all the tested pathogenic yeast strains except *C. glabrata* ZIM 2382 (112).

In another study, seven human breast milk-isolated LAB were characterized for their auto-aggregation, and co-aggregation potential with pathogenic *Shigella flexneri* and *Enterococcus faecalis*. Among the isolates, *Levilactobacillus gastricus* BDUMBT09 (MT774596), *L. brevis* BDUMBT11 (MW785062), *L. brevis* BDUMBT08 (MT673657), *L. casei* BDUMBT13 (MW785178), and *L. casei* BDUMBT12 (MW785063) demonstrated aggregation potential which increased with further incubation whereas *L. paracasei* BDUMBT10 (MT775430) and *Brevibacillus brevis* M2403 (MK371781) did not show such potential. Interestingly, all the isolates demonstrated better co-aggregation with *Enterococcus faecalis* as compared to *Shigella flexneri* (113). LAB isolates from human colostrum have been investigated for their bioprophylactic and biotherapeutic potential in another recent study. It reported *Streptococcus salivarius* CP163 and *S. salivarius* CP208 exhibit a higher anti-cancer potential among the 218 isolates. Both these probiotic bacteria displayed potential for auto-aggregation and co-aggregation with pathogens *Salmonella enteritidis* and *E. coli* (114).

The antibacterial potential of *Lactiplantibacillus plantarum* ATCC 1058 was evaluated by using *in vitro* simulated wound fluid (SWF) and *in vivo* wound models infected with two main burn wound-associated pathogens, i.e., *Pseudomonas aeruginosa* or *Staphylococcus aureus*. Results concluded both these pathogenic bacteria were susceptible to the *L. plantarum-*produced antimicrobial compounds and co-aggregation (115). Luan et al. evaluated the antimicrobial potential of *Lactobacillus curvatus* BSF206 and *Pediococcus pentosaceus* AC1-2 against the oral pathogen *Streptococcus mutans* (116). Both probiotic strains exhibited auto-aggregation and co-aggregation potential, these properties facilitate strains to adhere and colonize gingival epithelial cells and impede carcinogenic *S. mutans* from oral tissues. Moreover, the study reported that *L. curvatus* BSF206 and *P. pentosaceus* AC1-2 efficiently prevented *S. mutans* biofilm by downregulating the expression of key genes *ftf*, *brpA, gtfA*, and *gtfB* associated with biofilm formation (116).

### Maintenance of barrier integrity

The gut-mucosal barrier consists of the mucus layer, IECs, lamina propria, i.e., loose connective tissue containing the immune cells at the sub-epithelial level, and intestinal microbiota. Probiotics positively modulate all these levels and thereby inhibit gut barrier permeability (117, 118); “leaky gut” is associated with the pathogenesis of several disorders (119, 120). Different underlying mechanisms have been proposed through which probiotics induce favorable modulations to maintain gut barrier integrity (121); one such mechanism includes probiotic bacteria competing for the cellular adhesion sites. The enteropathogens must adhere to these epithelial adhesion sites in the gastrointestinal tract (GIT) to colonize effectively (122) but the probiotic bacteria efficiently adhere to these sites thus acting as “colonization barriers” thereby effectively inhibiting the pathogens from adhering to the IECs (123, 124). A study reported the use of *Lactobacillus rhamnosus* strain GG and *Lactobacillus plantarum* 299 v to demonstrate this effect. Both of these strains were able to prevent adherence of *Escherichia coli* to human colon cells (125).

The probiotics use several other mechanisms for the maintenance of the gut mucosal barrier (124, 126) including improved trans-epithelial electric resistance (TEER), increase in the levels of butyrate, upregulation of tight-junction (TJ) proteins (ZO-1, occludin, and claudin-1), elevated mucus secretion (by upregulation of MUC1, MUC2, and MUC3 in epithelial cells of colons) and as well as modulation of gut microbiota (127). These physiological changes are brought by molecular effectors secreted by probiotic bacteria, for instance, *L. plantarum* produces 10-hydroxy-cis-12-octadecenoic acid (HYA) which has been shown to repress the downregulation of ZO-1 (zonula occludin), occludin, and claudin-1 and TJ permeability induced by TNF-α and interferon-gamma (IFN-γ) *via* regulation of TNF receptor 2 expression by the G-protein-coupled receptor (GPR)-40/mitogen-activated kinase (MEK)/extracellular-signal-regulated kinase (ERK) dependent pathway (127).

The potential role of *Bifidobacterium dentium* N8 in protecting the gut barrier was examined by using human colorectal adenocarcinoma cells (Caco-2) as a model of the intestinal epithelial barrier. Lipopolysaccharide (LPS) was used to increase the permeability of the cell line. *B. dentium* N8 suppressed the permeability of Caco-2 and elevated the TEER by upregulating the expression of TJ proteins (ZO-1, occludin, and claudin) and downregulating the expression of IL-1β, IL-6, and TNF-α. Thus, attenuating inflammation and improving the gut barrier integrity (128). Another study reported the role of surface-layer proteins (Slps) from different *Lactobacillus* strains in maintaining the robustness of the gut barrier. Four Slps significantly prevented the reduction of TEER and TNF-α-induced permeability in Caco-2 monolayers; interestingly, TNF-α-induced downregulation of ZO-1 and occludin was also ameliorated. Moreover, these four Slps also attenuated TNF-α-induced elevated levels of IL-8 and NF-κB activation (129).

### Anti-inflammatory potential

Many studies have demonstrated that probiotics regulate inflammatory pathways, stimulate the expression of immune-related genes, and modulate the levels of immunological markers (130, 131). Recent studies have investigated the molecular mechanisms through which probiotics induce an anti-inflammatory response. One such study established a protective mechanism with breastmilk/probiotic use in necrotizing enterocolitis (NEC) (132); it is a serious gastrointestinal disease in premature infants caused by invasion of the pathogenic bacteria, the subsequent inflammation in the colon expedites to perforation and gut leakage, which leads to overwhelming infection and death. Whilst prevention is challenging, the best protection comes from the ingestion of maternal-expressed milk along with probiotics (133, 134). The study investigated the anti-inflammatory potential of secretions produced by *Bifidobacterium longum* subsp. *infantis* ATCC 15697 *via* ultra-high-performance liquid chromatography-tandem mass spectrometry (UHPLC-MS/MS). Indole-3-lactic acid (ILA), a *B. infantis*-secreted metabolite of breastmilk tryptophan was identified as an anti-inflammatory molecule and its potency was further investigated in a human immature small intestinal cell line, necrotizing colitis enterocytes, and mature enterocytes. ILA induced anti-inflammatory response *via* interacting with transcription factor aryl hydrocarbon receptor (AHR) to repress the IL-1β-induced transcription of IL-8 (132).

Inflammatory bowel diseases (IBD) arise from a wide spectrum of disorders that are marked by gut dysbiosis, chronic intestinal inflammation, mucosal ulceration, and ultimately loss of intestinal function (135, 136). Recent advances in research reveal a bidirectional relationship between gut dysbiosis and disease progression (136) therefore, current studies are focused on identifying novel therapies to modulate gut microbiota; one such study investigated the beneficial role of *Bifidobacterium bifidum* BGN4 as a probiotic (live) or parabiotic (heat or lysozyme treated) in alleviating dextran sulfate sodium (DSS)-induced colitis in mice (137). It was observed that the lysozyme-treated *B. bifidum* BGN4 group demonstrated better-preserved integrity of the intestinal barrier *via* upregulation of TJ protein expression probably by stimulating the NLRP-6/caspase-1/IL-18 signaling pathway. Moreover, the lysozyme treated group demonstrated immune tolerance by upregulating the expression of forkhead box P3 (FOXP3) and downregulating the pro-inflammatory molecules including IL-1β, cyclooxygenase-2 (COX-2), T-bet, and inhibitor of kappa light chain gene enhancer in B cells, alpha I-kappa-B-alpha (IκB-α), thereby exerting a preventive effect against DSS-induced colitis (137). Similarly, Dong et al. used *Pediococcus pentosaceus* CECT 8330 (138), while Li et al., used *Lactobacillus acidophilus* ATCC 4356 (139) and reported that the probiotic strains exhibited high anti-inflammatory potential and could be used to restore gut dysbiosis, preserve gut barrier integrity, and ameliorate intestinal inflammation.

**FIGURE 2:**
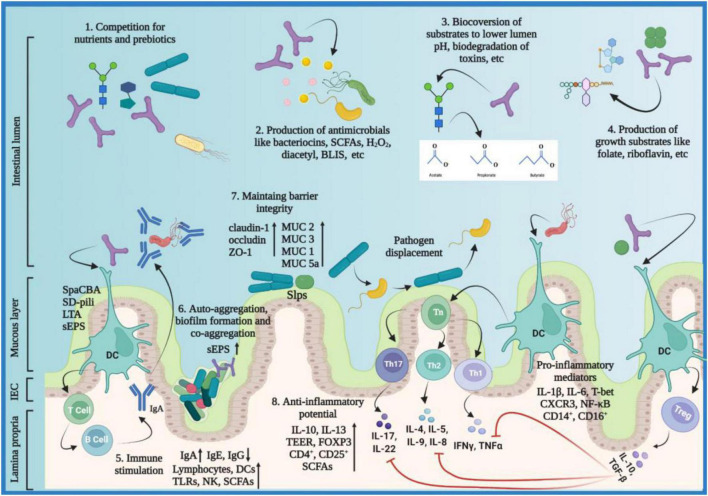
Probiotics action modality. Probiotics employ various mechanisms to exert their beneficial effects on the host including: (1) probiotics compete pathobionts and pathogens for microbial resources needed for growth and metabolism, e.g., acquisition of monosaccharides; (2) probiotics inhibit pathogens by producing antimicrobials, e.g., SCFAs, bacteriocins, antibiotics, microcins, etc.; (3) probiotics metabolize substrates into useful products, e.g., organic acids and volatile fatty acids; (4) probiotics produce growth substrates for beneficial microbiota and the host, e.g., folate and riboflavin; (5) probiotics induce immunomodulatory responses either by direct contact or surface molecules like SpaCBA, SD-pili, LTA, and sEPS; immune stimulation favors elevated expression of IgA and SCFAs in pathogenic infections and decreased expression of IgE and IgG in allegro-inflammatory responses *via* stimulating DCs, TLRs, NK cells, and lymphocytes; (6) sEPS facilitate probiotics to auto-aggregate and form protective biofilms and, co-aggregate with pathogens to prevent them from colonizing the host epithelial surfaces; (7) probiotics maintain barrier integrity by regulating TJ proteins (claudin-1, occludin, ZO-1) and by mucus formation *via* elevated expression of MUC 1,2,3 and 5a; Slps facilitate attachment of the probiotic strains to the gut epithelium; the colonization of pathogens is inhibited through pathogen displacement; (8) probiotics reduce inflammation by upregulating anti-inflammatory and downregulating pro-inflammatory mediators; CXCR3, C-X-C Motif Chemokine Receptor 3; BLIS, bacteriocin-like inhibitory substances; DC, dendritic cell; FOXP3, forkhead box p3; H_2_O_2_, hydrogen peroxide; IFN-γ, interferon-gamma; Ig, immunoglobulin; IL, interleukin; LTA, lipoteichoic acid; MUC, Mucin; NF-κB, nuclear factor-kappa light chain enhancer of activated B cells; NK, natural killer; SCFA, short chain fatty acids; SD-pili, sortase-dependent pili; sEPS, surface exopolysaccharides; Slps, surface-layer proteins; spaCBA, heterotrimeric pili complex made of protein subunits spa C, spa B and spa A; TEER, trans-epithelial electrical resistance; TGF-β, transforming growth factor-beta; Th, T-helper; TJ, tight junction; TLR, toll-like receptor, TNF-α, Tumor necrosis factor-alpha; Treg, T regulatory. The figure was drawn with BioRender.

Tungsanga et al. investigated the protective role of *Lactobacillus rhamnosus* 34 on enterocyte integrity and renal fibrosis. The study was carried out using 5/6-nephrectomy (5/6-Nx) mice, Caco-2 enterocytes, and HK2 proximal tubular cells. *L. rhamnosus* 34 demonstrated positive effects in 5/6-Nx mice evident by a significant decrease in GDUT (gut-derived uremic toxin) index including endotoxins, trimethylamine-N-oxide (TMAO), and indoxyl sulfate (IS) and reduction in renal injuries exhibited by lower serum creatinine and proteinuria levels in kidney fibrosis area and decrease in serum TNF-α when compared to 5/6-Nx controls. Furthermore, the *L. rhamnosus* 34-conditioned media downregulated the expression of IL-8, NF-κB and improved TEER value in Caco-2 enterocytes, and ameliorated the expression of IL-6, TNF-α, and collagen in HK2 proximal tubular cells, supporting *L. rhamnosus* 34 as a potential biotherapeutic for chronic kidney disease (CKD) (140). Recent advancements in multi-omics techniques revealed that gut dysbiosis is related to CKD and this phenomenon exacerbates in hemodialysis pediatric and adult patients. The gut dysbiosis-induced systemic inflammation contributes to cardiovascular diseases, like atherosclerosis. Additionally, the relation between gut dysbiosis and CKD is found to be bidirectional, a positive shift in the gut microbiome corresponds to a decrease in circulatory GDUT (141–143). Choi et al. investigated the potential of *Bifidobacterium bifidum* BGN4 and *Bifidobacterium longum* BORI in reducing the inflammation and inflammation-associated cardiovascular risks in hemodialysis patients. The study concluded that there was a significant increase in fecal SCFAs and a significant decrease in IL-6 and serum calprotectin levels post-probiotic treatment. The anti-inflammatory potential of the *Bifidobacteria* strains was characterized by a significant decrease in the percentages of proinflammatory monocytes CD14^+^ and CD16^+^, and a significant increase in anti-inflammatory T regulatory cells CD4^+^ and CD25^+^. The study concluded that both *Bifidobacteria* strains possess the potential to mitigate systemic inflammation and cardiovascular diseases in hemodialysis patients (144). The probiotic action modality with associated molecular mediators is summarized below (Figure 2).

## What factors govern the efficacy of probiotics in given circumstances?

A wide spectrum of probiotics labeled with various health claims is available in the market. The production of probiotics has progressively surged as they are continuously prescribed by clinicians as biotherapeutics and consumed by the general public as over-the-counter bioprophylactics. Consequently, the global probiotic market was estimated to be valued at USD 61.1 billion in 2021 and is projected to reach USD 91.1 billion by 2026 (145). However, the ever-expanding probiotic product base is often mislabeled by the industry and misunderstood by the consumers. Probiotic manufacturing must avoid inconsistencies by encompassing correct labeling of the product, safety, and potency (146, 147); furthermore, the probiotic strains must endure the manufacturing processes and exposure to environmental factors to remain viable and retain the ability to colonize the gastrointestinal tract (148). Cell encapsulation improves the resistance of probiotic strains to adverse conditions (149, 150).

**FIGURE 3:**
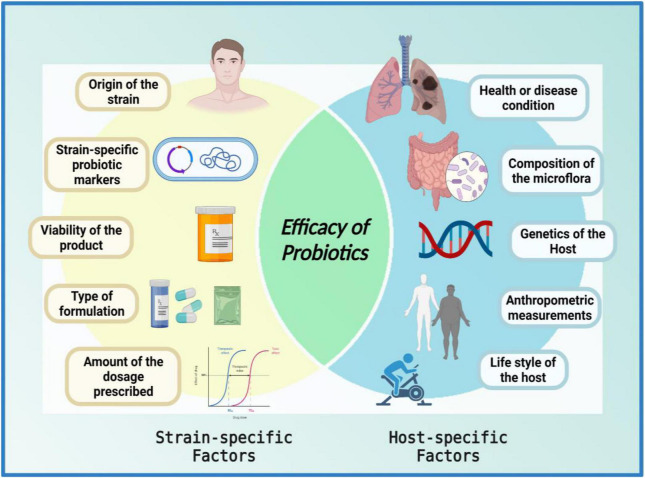
Venn diagram of factors affecting the efficacy of probiotics. The efficacy of a probiotic is a result of the interplay between numerous strain-specific and host-specific factors. The figure was drawn with BioRender.

Despite having been conceived as homogenous mixtures of beneficial microorganisms, probiotic properties are both strain-specific and disease-specific (151), i.e., each probiotic strain has its unique repertoire of functional genes. It is imperative to understand that when it comes to the functional capacity of probiotics, a given preparation could not be all-encompassing in a given condition (7, 8, 44, 152–154). For a successful treatment, the selection of an appropriate probiotic must be based on probiotic-specific and host-specific factors (Figure 3). The probiotic-specific factors include: origin of the strain, strain-specific probiotic genetic markers, type of formulation, viability of the strain, and amount of dosage prescribed; while the host-specific factors include: type of disease or indication, composition of the gut microflora, diet, age, anthropometric measurements, and lifestyle of the host (7, 44, 151).

*“The efficacy of a probiotic is a measure of its ability to adapt to the host environment and exert its unique health-promoting effects; furthermore, the interplay between numerous strain-specific and host-specific factors conforms the efficacy of probiotics in a given circumstance.”*

## What criteria should be met for the formation of probiotics intended for human use?

The formulation of human probiotics requires evaluation of the health-promoting microorganisms by a global standard that is summarized as follows:

• As per World Health Organization (WHO)/Food and Agriculture Organization (FAO) recommendations, the origin of a probiotic strain must correlate to its site of action in the host; only isolates from human origin, viz. small intestine, large intestine, and breast milk have been approved to formulate probiotics for human use (155).
• The isolates must be carefully characterized and examined for their beneficial effects to be considered as a probiotic (44, 155).
• As per European Food Safety Authority (EFSA) recommendations, all microorganisms intended for human use must acquire genus, species, and strain levels of taxonomic identification (7, 8, 44, 45).
• Most commonly used probiotics are Lactic acid bacteria (LAB). They are identified as Generally Recognized as Safe (GRAS) by Food Drug Administration (FDA) and have Qualified Presumption of Safety (QPS) status by EFSA. Despite that, any strain intended as a probiotic for human use must undergo a rigorous safety assessment (44, 155–158).
• Moreover, as per recent EFSA guidelines, Whole-Genome Sequence (WGS)-based analysis of candidate strains is required for the formulation of probiotics (45).
• For the selection of a putative probiotic strain, its behavior under simulated GIT conditions has to be investigated. The probiotic candidates must tolerate acid and bile stress and osmotic variations to survive gastrointestinal transit (159).
• Finally, after satisfying the above-mentioned criteria, the health benefits proffered by probiotic candidates need validation through preclinical trials followed by double-blind and randomized human clinical trials (152).
• The recommended probiotic dose is between 10^8^ (hundred million) and 10^11^ (hundred billion) viable colony forming units (CFU/mL/g) per day (152).

## Why do certain probiotics perform inadequately in pre-clinical and clinical trials?

**FIGURE 4:**
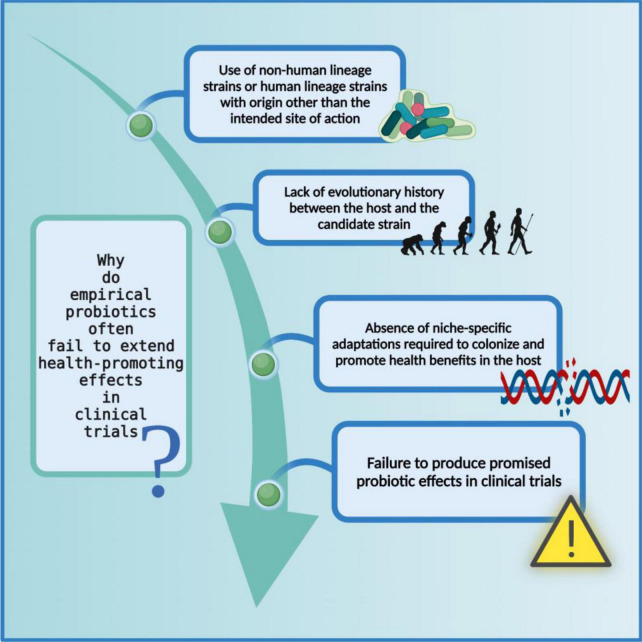
Limitations of non-human lineage strains as probiotics. The probiotic formulations based on non-human associated strain often fail to produce health-promoting effects in clinical trials possibly because they did not co-evolve with their respective host and consequently do not carry niche-specific genetic modifications. The figure was drawn with BioRender.

Irrespective of the WHO/FAO outlined recommendations, a plethora of scientific literature is based on identifying non-human lineage probiotic strains intended for human use. It not only taints the efficaciousness of probiotics but also causes discord between the industry, medical, and scientific communities. The *in vitro* probiotic properties displayed by non-human lineage strains often fail to extend in clinical trials due to the difference between their source of isolation and site of action (160); the ecological history of a probiotic strain, although often overlooked, plays a significant role in determining the fate of that strain i.e., to reach and then to survive at the required site of action in the host (161–164) (Figure 4). To understand the full extent of the dynamics between the source of isolated strain and the rate of success of the treatment, let us revise the concept of autochthonous vs. allochthonous strains. The autochthonous microbial strains are native to the host environment whereas the allochthonous microbial strains are non-native to the host environment. Some of the autochthonous and allochthonous strains that are frequently isolated from the human gastrointestinal tract and are used in probiotic formulations are listed below (165–171) (Table 3).

**TABLE 3:** Some frequently human gut-isolated allochthonous and autochthonous strains that are commonly used as probiotics with revised nomenclature.

Strain association	Strain name	References
Allochthonous strains	*Lactobacillus acidophilus* *Lacticaseibacillus casei* (previously *Lactobacillus casei*) *Limosilactobacillus fermentum* (previously *Lactobacillus fermentum*) *Lactobacillus delbrueckii* *Levilactobacillus brevis* (previously *Lactobacillus brevis)* *Lactobacillus johnsonii* *Lactiplantibacillus plantarum* subsp. *plantarum* (previously *Lactobacillus plantarum*) *Lacticaseibacillus rhamnosus* (previously *Lactobacillus rhamnosus*) *Lacticaseibacillus paracasei* subsp. *paracasei* (previously *Lactobacillus paracasei*)	(32, 165, 171)
Autochthonous strains	*Limosilactobacillus reuteri* (previously *Lactobacillus reuteri*) *Ligilactobacillus ruminis* (previously *Lactobacillus ruminis*) *Lactobacillus gasseri* *Ligilactobacillus salivarius* (previously *Lactobacillus salivarius*) *Limosilactobacillus mucosae* (previously *Lactobacillus mucosae*)	(32, 165, 166)

Contributing to the significance of the ecological history of probiotics, a study reported the colonization potential of two strains *Lactobacillus mucosae* FSL-04 and *Lactobacillus reuteri* ATCC PTA 6475, both autochthonous to the human GIT compared with *Lactobacillus acidophilus* DDS-1, allochthonous to human GIT. The study concluded that autochthonous strains were established more efficiently in the human GIT than the allochthonous strain. However, when consumption was discontinued, all three strains became undetectable on the eighth day, highlighting the fact that probiotics persist in the gut only transiently (172). The potential of the probiotic strains to colonize the human GIT is dependent on the host microbiota. The colonization-resistant microbiome hinders the persistence of probiotic strains as opposed to the colonization-permissive microbiome (7, 173–175). Furthermore, studies have shown that even the autochthonous strains of one individual may not be able to colonize the GIT of another individual due to personalized differences (153).

*“The rate of success of a probiotic intervention α establishment of a strain in the microecological niche of the host + production of microbial metabolites that positively influence the physiological pathway of the host and/or their gut microbiota.”*

## Why should host-adapted strains be favored for probiotic formulations?

Indifferent to the autochthonous vs. allochthonous origin of a probiotic isolate, the ability of an isolate to adapt to a specific ecological niche is accomplished by genome specialization. It is a process whereby, niche-specific phenotypic fitness is acquired through selective genome decay of unutilized genes and enrichment of the genes that facilitate adaptability in the respective habitat. Therefore, individual probiotic strains isolated from the same habitat carry the same niche-specific genetic signatures. This has been validated for *Lactobacillus jensenii*, and *Lactobacillus gasseri* both isolated from the human vagina as well as for *Lactobacillus reuteri* isolates from the GI tract of different vertebrates (43, 164). Similarly, *Lactobacillus* species including *L. plantarum*, *L. rhamnosus*, *L. paracasei*, and *L. casei* although not considered autochthonous in a classical sense, possess niche-specific adaptations to oral cavity and gut ecosystems that enable them to persist transiently (162). Studies have shown that host-adapted *Lactobacilli* strains show higher ecological fitness in their respective host (163, 176). Since host-adapted strains carry niche-specific genomic modifications, they display higher ecological fitness as opposed to the strains that do not share an evolutionary history with the host. The higher ecological fitness enables higher metabolic activity and higher resistance to enteropathogens (163). Also, host-adaptive strains establish immunotolerant profiles in host gut mucosa (177). This has been reported for *L. reuteri* strains as the human lineage autochthonous *L. reuteri* strain displayed anti-inflammatory effects while the poultry lineage *L. reuteri* strain displayed immune-stimulatory effects in human myeloid cells (161, 176).

**FIGURE 5:**
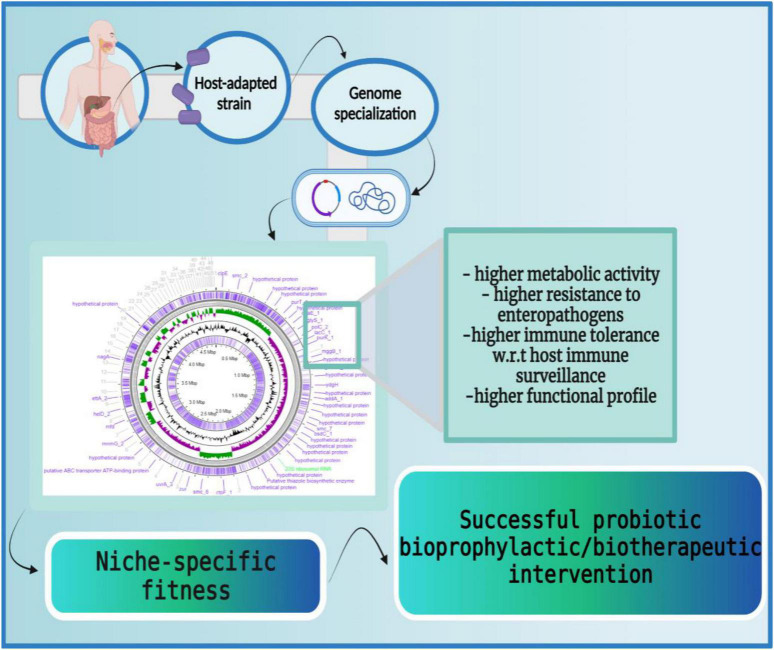
Host-adapted strains harbor niche-specific phenotypic fitness. The host-adapted microbial strains carry niche-specific genetic signatures which enable them to adapt to the host ecosystems efficiently. These genetic modifications enable higher metabolic activity (larger carbohydrate utilization cassettes, production of β-galactosidases, etc.), higher resistance to enteropathogens (production of lactic acids, synthesis of secondary metabolites, etc.), higher immune tolerance (induction of anti-inflammatory cytokines like IL-10, IL-13, etc.), and higher functional profiles (production of bile salt hydrolases to endure bile salts, accumulation of ATP synthesizing cassettes for conditional respiration, etc.). Thus, host-adapted microbial strains possess a large repertoire of niche-specific genes that facilitate their persistence in the host. The figure was drawn with BioRender.

Supporting the above-mentioned studies, Doron et al. reported that the attempt spanned over many years to colonize dairy-isolated *Lactobacillus* in the human gastrointestinal tract (GIT) is futile. The dairy-isolated *Lactobacillus* strains including *L. acidophilus, L. casei*, and *L. bulgaricus* do not harbor the necessary biological characteristics to successfully establish in the human host and thereby, are unlikely to demonstrate any beneficial effects (178). Similarly, Wong et al., reviewed differences in the physiological attributes (i.e., metabolic capabilities), and genetic attributes (i.e., comparative and functional genomics) of human residential and non-human residential *Bifidobacteria* strains (179). The study concluded that human residential strains possess better adaptive health attributes for their host (178) e.g., human infant-derived *Bifidobacteria longum* subsp. *infantis* has a gamut of glycoside hydrolases (GHs) and ABC transporters required for degrading human milk oligosaccharides (HMOs) and *fol* gene clusters for folate biosynthesis as opposed to non-human-associated *Bifidobacteria* (180) (Figure 5).

*“Thus, the ecological and evolutionary characteristics of a probiotic isolate are a measure of its functionality.”*

## Strategic formulation of target-based probiotics for human use

**FIGURE 6:**
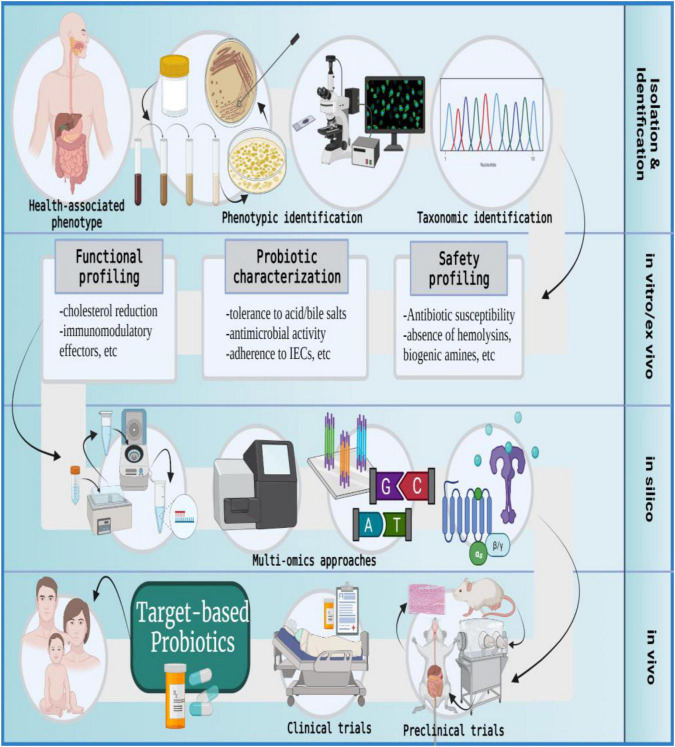
The formulation of target-based probiotics. The formulation of target-based probiotics is based on understanding the action modality of probiotic strains *via* a multi-omics approach and then tailoring a probiotic formulation that produces molecular effectors to ameliorate a specific disease or indication; IECs, intestinal epithelial cells. The figure was drawn with BioRender.

As has been discussed earlier, non-human lineage probiotics often fail to extend the *in vitro*-exhibited beneficial effects in clinical trials (160). We shall now discuss the use of host-adapted microbial isolates in the formulation of target-based probiotics intended for human use (163, 176) (Figure 6). As per standard guidelines, the source of isolation of a probiotic strain must correlate with its intended site of action. For instance, if the attenuation of gut dysbiosis is intended then the source of isolation should be the human GI tract. Similarly, if treatment for bacterial vaginosis or periodontitis is intended then corresponding probiotics must be formulated using vaginal or oral isolates, respectively (44).

The metamorphoses of commensal microbiota into target-based probiotics involves the following procedure: The samples are collected from healthy individuals, and microbial strains are isolated using a specific growth medium. The isolates are subjected to phenotypic characterization and selected candidates are taxonomically identified on the genus-species-strain level (44, 45). The *in vitro* and *ex vivo* approaches evaluate the candidate probiotics in terms of safety and functionality. The *in silico* analysis uses multi-omics (genomics, transcriptomics, proteomics, and metabolomics) approaches to further identify molecular effectors (microbial metabolites) produced by the selected probiotic candidate that can potentially modify host or microbial pathways to promote health phenotype or ameliorate disease conditions. Post *in silico* analysis, the *in vivo* studies entail validation of proposed probiotic effects by using gnotobiotic animal models with metabolic, immune, microbial, or neuronal distinctions. Finally, the success of clinical trials culminates in the development of target-based probiotics for human use (10, 31, 152, 181–186).

## Conclusion

Probiotics have gained substantial amount of attention over the past few decades and with the advancements in multi-omics techniques, we now understand better than ever, the role of probiotics in maintaining gut microbiota and health phenotype. A wide spectrum of probiotic products is available in the market and the probiotic industry is rejoicing in its exponential boom. However, the development of probiotics must be medical interest-driven rather than commercial interest-driven. Despite a large product base, there is heterogeneity between the claimed probiotic benefits in scientific literature and the efficacy of probiotics in clinical trials which can be resolved by understanding that the efficacy of a probiotic intervention depends on both the strain-specific and host-specific factors. The formulation of human probiotics requires a strict evaluation of candidate strains by a global standard. The harmony between the probiotic source of isolation and its intended site of action determines the success of a subsequent probiotic intervention. The host-adapted probiotic strains are likely to harbor selective genetic modifications that facilitate their niche-specific phenotypic fitness in the respective host. The evolutionary and ecological history of a probiotic strain, albeit often overlooked, heralds a significant outcome; ergo, the formulation of probiotics using host-adapted strains with known molecular effectors would serve as ideal candidates for target-based bioprophylactic and biotherapeutic interventions to maintain health and ameliorate diseased phenotypes.

## Author contributions

MId and SG: conceptualization. MId: illustrations and writing—original draft preparation. MIm, RZ, NA, MA, MT, and AF: validation. MId, MIm, MA, OO, and SG: formal analysis. MIm, SG, RA, and AF: investigation. AA, TR, and OO: resources. MA, TR, RA, and AA: data curation. MId, MIm, OO, and MA: writing—review and editing. All authors contributed to the article and approved the submitted version.

## Abbreviations

ABC: ATP-binding cassettes
AHR: aryl hydrocarbon receptor
AKT: protein kinase B
BALF: bronchoalveolar lavage fluid
BLIS: bacteriocin-like inhibitory substances
CBER: Center for Biologics Evaluation and Research
CFU: colony forming units
CKD: chronic kidney disease
COVID-19: coronavirus disease of 2019
COX-2: cyclooxygenase-2
CXCR3: C-X -C Motif Chemokine Receptor 3
DC: dendritic cell
DSS: dextran sulfate sodium
EFSA: European Food Safety Authority
EPO: eosinophil peroxidase activity
EPS: exopolysaccharides
ERK: extracellular-signal-regulated kinase
FAD: flavin adenine dinucleotide
FAO: Food and Agriculture Organization
FDA: Food and Drug Administration
FMN: flavin mononucleotide
FOXP3: forkhead box p3
GABA: gamma-aminobutyric acid
GDUT: gut-derived uremic toxin
GH: glycoside hydrolase
GIT: gastrointestinal tract
GPR: G-protein -coupled receptor
H_2_O_2_: hydrogen peroxide
HMO: human milk oligosaccharides
HYA: 10-hydroxy-cis-12-octadecenoic acid
IBD: inflammatory bowel disease
IEC: intestinal epithelial cell
IFN- γ: interferon-gamma
Ig: immunoglobulin
I κ B- α, inhibitor of kappa light chain gene enhancer in B cells: alpha I-kappa -B-alpha
IL: interleukin
ILA: indole lactic acid
IS: indoxyl sulfate
ISAPP: International Scientific Association for Probiotics and Prebiotics
LAB: lactic acid bacteria
LTA: lipoteichoic acid
MALDI-TOF MS: matrix-assisted laser desorption ionization–time-of-flight mass spectrometry
MEK: mitogen-activated kinase
MUC: Mucin
MyD88: Myeloid differentiation primary response 88
NEC: necrotizing enterocolitis
NF- κ B: nuclear factor-kappa light chain enhancer of activated B cells
NK: natural killer
NLRP3: NOD-like receptor family pyrin domain containing 3
PI3K: Phosphoinositide 3-Kinase
pABA: para-aminobenzoic acid
PCR: polymerase chain reaction
PMB: probiotics-mediated bioconversion
PRRs: Pattern recognition receptors
SCFA: short-chain fatty acids
SD-pili: sortase-dependent pili
sEPS: surface exopolysaccharides
Slps: surface-layer proteins
spaCBA: heterotrimeric pili complex made of protein subunits spa C spa B and spa A
TEER: trans-epithelial electrical resistance
TGF- β: transforming growth factor-beta
Th: T-helper
TJ: tight junction
TLR: toll-like receptor
TMAO: trimethylamine-N-oxide
TNBS: trinitrobenzene sulfonic
TNF- α: Tumor necrosis factor-alpha
Treg: T regulatory
UHPLC-MS/MS: ultra-high-performance liquid chromatography-tandem mass spectrometry
WGS: Whole-genome sequence
WHO: World Health Organization.
